# Mast Cell Clonal Disorders: Classification, Diagnosis and Management

**DOI:** 10.1007/s40521-016-0103-3

**Published:** 2016-11-08

**Authors:** Merel C. Onnes, Luciana K. Tanno, Joanne N. G. Oude Elberink

**Affiliations:** 1 Department of Allergology, University Medical Center Groningen, University of Groningen, and Groningen Research Institute for Asthma and COPD, Groningen, The Netherlands; 2 Hospital Sírio Libanês, São Paulo, Brazil; 3 University Hospital of Montpellier, Montpellier, and Sorbonne Universités, UPMC Paris 06, UMR-S 1136, IPLESP, Equipe EPAR, 75013 Paris, France

**Keywords:** Mastocytosis, Mast cell clonal disorders, Allergy, Anaphylaxis, Osteoporosis, Tyrosine kinase inhibitors

## Abstract

Mast cell clonal disorders are characterized by the clonal proliferation of pathological mast cells as a result of somatic mutations in the KIT gene, most commonly the D816V mutation. Accumulation and degranulation of these cells causes a wide variety of symptoms. Mast cell clonal disorders can be divided into mastocytosis and monoclonal mast cell activation syndrome, depending of the level of clonality. The severity of mastocytosis varies from an indolent variant with a good prognosis, to an aggressive condition with short life expectancy. Diagnosis is based on demonstration of clonality and accumulation in the skin and in extracutaneous tissues. Treatment is highly individualized, and is based on the severity of the condition. Treatment of patients with indolent systemic mastocytosis is aimed at reducing symptoms, using histamine H1 and H2 receptor antagonists as a starting point. In addition, associated conditions such as osteoporosis must be treated. Treatment of advanced systemic mastocytosis is aimed at reducing mast cell load through cytoreductive therapy. The choice of such therapy depends on the KIT mutational status. Though currently there is no curative treatment available, promising new therapies such as midostaurin are emerging that have demonstrated success in reducing symptoms and improving quality of life.

## Introduction

Mast cells (MCs) are important effector cells of the immune system, best known for their role in allergies and anaphylaxis. They also play an important protective role, and are involved in wound healing, angiogenesis, immune tolerance, pathogen defence and blood–brain barrier function.

MCs are characteristically found surrounding blood vessels and nerves, but are especially prominent near the boundaries between the outside world and the internal milieu, e.g. in the skin, lungs and gastrointestinal tract. MCs are distinguished by their high content of granules, containing pro-inflammatory and vasoactive mediators.

MC disorders may involve abnormal reactivity with normal MC numbers and/or excessive proliferation of MCs, known as mastocytosis.

Mastocytosis is the most important MC clonal disorder. It is a rare condition relevant to many fields of medicine. The condition is characterized by the clonal proliferation of pathological MCs. The clinical presentation is highly heterogeneous. Features of systemic mastocytosis are caused by the accumulation of clonally derived MCs in different tissues and by the abnormal reactivity, causing degranulation of the MC. Proper treatment of this condition is essential and requires a multidisciplinary approach. Thus far, there is no curative treatment for mastocytosis. However, over the last few years, new methods for treating this condition have emerged. In this article we review the current views on the pathophysiological mechanisms, classification, diagnosis and management of MC clonal disorders.

## (Patho)physiology

MCs are immunological cells derived from progenitor cells in the bone marrow, which mature in diverse peripheral tissues [1]. In allergic reactions, activation of MCs occurs after the MC is exposed to an antigen that cross-links allergen-specific IgE, which is already bound to the high-affinity Fc epsilon receptor 1 (FcεR1) on the MC. However, they can be activated by many triggers other than allergens, such as extreme temperatures and emotional stress [2•].

In normal MCs, stem cell factor (SCF) binds to the CD117 transmembrane tyrosine kinase receptor, which is encoded by KIT. This receptor regulates MC growth, migration and survival [2•]. In MC clonal disorders, patients show somatic mutations in enzyme and receptor genes involved in regulating MC activity [3]. These mutations cause reduced apoptosis and increased proliferation of MCs, eventually leading to MC accumulation and easily triggered activation of MCs [4]. The most common mutation is the D816V mutation in the tyrosine kinase KIT. This mutation is detected in up to 90 % of adult patients with systemic mastocytosis, and results in a tyrosine kinase receptor in a constantly activated state [3, 5].

## Clinical presentation

Symptoms in patients with monoclonal MC disorders are caused by the accumulation and degranulation of MCs. MC accumulation results in infiltration of various tissues, such as the skin and the bone marrow, which may lead to symptoms such as typical skin lesions in the form of urticaria pigmentosa or osteolytic lesions in the bone. Degranulation of MCs can cause MC activation (MCA) [2•]. Systemic MC activation (MCA) is characterized by three main findings: first, the presence of typical signs and symptoms, e.g. flushing, pruritus, urticaria, nasal congestion and hypotension; second, a substantial and transient increase in one or more MC-derived mediators (e.g. tryptase) in biological fluids after activation; and third, an objective response of symptoms to treatment with histamine receptor blockers or MC-targeting agents [6].

## Classification

Mastocytosis is a clonal MC disorder, and can be subdivided into cutaneous mastocytosis (CM), systemic mastocytosis (SM) and MC tumours. Criteria for diagnosing mastocytosis can be found in Table 1. SM may present with or without cutaneous involvement. In CM, only criteria for mastocytosis in the skin (MIS) are met. In SM, the major criterion—the presence of multifocal MC clusters—and one minor criterion, or three minor criteria must be fulfilled. Minor criteria consist of large proportions of MCs with abnormal morphology, detection of a D816V KIT mutation, MC expression of CD2 or CD25, and elevated tryptase levels in the blood.

**Table 1:** SM and MIS criteria, based on Valent et al. [7]

Criteria for systemic mastocytosis	
Major	Multifocal dense aggregates of >15 MC/cluster in the bone marrow (BM) or in other extracutaneous tissues (EC)
Minor	>25 % of MC have an abnormal morphology in BM or are spindle-shaped in EC infiltrates
	A KIT mutation at codon 816 in EC, preferably bone marrow
	CD 2 and/ or CD25 expression of MC in BM or other EC
	Serum tryptase > 20 ng/mL
*The diagnosis SM requires at least 1 major and 1 minor criteria, or 3 minor SM criteria.*	
Criteria for mastocytosis of the skin (MIS)	
Major	Typical skin lesions or atypical lesions combined with a positive Dariers’ sign and exclusion of other skin diseases
Minor	“Mast cell aggregates of > 15 MC/cluster or monomorphic infiltrate with > 20 MC per high-power field”
	KIT D816V in lesional skin
*To diagnose CM at least 1 major and 1 minor MIS criteria are met and the criteria to diagnose SM are not met.*	

Adults more often suffer from SM, whereas children mainly suffer from CM. The typical maculopapular red or brown skin lesions in mastocytosis, urticaria pigmentosa (UP), are often localized on the thighs, axilla and the lower trunk, and may spread over a period of years. Mechanical stroking of the lesions usually produces wheals and reddening, which is known as Darier’s sign [8•]. The skin is involved in approximately 80 % of all mastocytosis cases [9].

Urticaria pigmentosa can be divided into two variants: a monomorphic variant with small maculopapular lesions, and a polymorphic variant with larger lesions of variable size and shape. This distinction has important prognostic implications [8•]. The monomorphic variant is typically seen in adult patients, whereas the polymorphic variant is more often observed in paediatric patients. Clinical observations suggest that the monomorphic variant in children often persists into adulthood, whereas the polymorphic variant may resolve around puberty. The differences between childhood and adult mastocytosis are listed in Table 2.

**Table 2:** Characteristics of typical adulthood-onset and childhood-onset mastocytosis as published by Hartmann et al. [8•]

	Adulthood-onset mastocytosis	Childhood-onset mastocytosis
Most frequent category of mastocytosis	ISM	CM
Typical course of the disease	Chronic	Temporary
Frequency of anaphylaxis (%)	50	<10
Typical tryptase level (μg/l)	>20	<20
Typical location of KIT mutation	Exon 17, most frequently KIT D816V	Exon 8, 9, 11 or 17, or absent
Most frequent type of cutaneous lesions	Maculopapular	Maculopapular
Typical morphology of maculopapular lesions	Monomorphic	Polymorphic
Typical size of maculopapular lesions	Small	Large
Typical distribution of maculopapular lesions	Thigh, trunk	Trunk, head, extremities

SM can be subdivided into various forms, the most prevalent being indolent systemic mastocytosis (ISM), characterized by a low rate of proliferation and a good prognosis [10]. Smouldering SM and aggressive SM (ASM) are more severe and are characterized by B (borderline benign) and C (consider cytoreduction) findings, respectively. B findings, e.g. tryptase levels higher than 200 ng/mL or bone marrow infiltration greater than 30 %, indicate organ involvement without dysfunction. C findings, e.g. bone marrow dysfunction or malabsorption due to GI infiltrates causing weight loss, indicate organ dysfunction [11].

Another variant of SM is SM with associated clonal haematological non-MC-lineage disease (SM-AHNMD). MC tumours consist of mast cell leukaemia (MCL) and mastocytoma. ASM, SM-AHNMD and MCL are also referred to as advanced systemic mastocytosis, and all have a more aggressive course and a poorer prognosis [12].

Over the years it has been noted that there are patients with a KIT mutation who do not meet the criteria for mastocytosis. These patients are classified under the term monoclonal mast cell activation syndrome (MMAS). MMAS patients fulfil a maximum of only one or two SM minor criteria. Symptoms of MMAS are similar to those of SM, but typical skin lesions are lacking. Treatment of MMAS is based on the patient’s symptoms; however, the principle of treatment is comparable to that for patients suffering from ISM.

In summary, monoclonal MC disorders encompass both mastocytosis and MMAS.

## Presentation

The presentation of patients with mastocytosis is heterogeneous. Variation can be found not only between the various subtypes, due to the different levels of severity of the condition, but also within subtypes. As mentioned above, symptoms are caused either by the release of mediators (e.g. anaphylaxis, flushing, abdominal cramping, pruritus and fatigue) or by MC infiltration (e.g. urticaria pigmentosa, portal hypertension, hypersplenism and malabsorption). Given the heterogeneous presentation, patients may experience little or no inconvenience related to their condition, or they may suffer from symptoms with a high impact on their quality of life (QoL). Recent studies have validated several QoL questionnaires for mastocytosis, identifying fatigue and fear of anaphylaxis as the symptoms causing the greatest inconvenience and impact on patient QoL [13•, 14•].

Anaphylactic reactions are a common feature in mastocytosis, occurring in approximately half of all patients [15]. Several factors may trigger anaphylactic reactions, including hymenoptera stings, alcohol consumption, drugs, general anaesthetics and contrast media. Hymenoptera stings, however, are the most common trigger for severe, life-threatening anaphylactic reactions, whereas food and medications generally elicit milder reactions. Approximately 4 % of patients with a history of hymenoptera sting anaphylaxis (HVAn) have CM or SM [16•]. Patients may develop HVAn at a later stage of their condition. The exact mechanism that evokes HVAn in patients with mastocytosis has not yet been elucidated, though some studies have identified risk factors for HVAn in mastocytosis patients. Individuals with a less differentiated gene expression of cells in the peripheral blood appear to have a lower risk of HVAn [17]. Dedifferentiated MCs are more prone to dysfunction, which could explain the finding that higher MC load reduces the risk of HVAn [18].

Osteoporosis is another common feature in patients with mastocytosis. Osteoporosis develops because of both the accumulation and degranulation of MCs in the bone. This can also lead to osteolytic lesions or even pathological fractures. The reported prevalence of osteoporosis in ISM patients is high, at 18 to 31 % [19]. Patients are often male and younger than the general osteoporotic population. A high prevalence of pathological fractures, 37 %, has been reported as well [20••].

## Diagnosis

For the detection or exclusion of the criteria for SM, bone marrow examinations are required. In order to avoid unnecessary bone marrow biopsies, skin biopsy and measurement of total tryptase in the serum and histamine metabolites in urine (MH, MIMA) are performed at baseline and after symptomatic events to assess the probability that SM is present. A study in which tryptase levels were measured in patients with suspected ISM showed that tryptase levels lower than 10 μg/L indicated a very low risk of ISM. The risk of ISM with tryptase levels >10 μg/L is dependent on the levels of methylhistamine (MH) and methylimidazole acetic acid (MIMA). If these are elevated, the risk of mastocytosis is high; if not, the risk is low [21].

If there is an indication for bone marrow examination, bone marrow biopsies should be examined histologically (e.g. for the presence of osteoporosis) and immunohistochemically, stained with antibodies against tryptase, CD117 and CD25. Bone marrow aspirates should be examined cytologically, using Wright-Giemsa and toluidine blue staining, and with flow cytometry. In flow cytometry, cells should be processed within 24 h. Minimum assessment of CD2, CD25, CD45 and CD117 is recommended. Unlike normal MCs, the MC of patients with SM express the surface markers CD2 and/or CD25 [16•]. In addition, KIT mutation analysis should be performed, especially to detect the presence of a D816V mutation. If this mutation is absent, other KIT mutations may be present. KIT mutation analysis can also be performed in other tissues, but its sensitivity is highest in the bone marrow [22–24].

## Treatment

Treatment of mastocytosis is largely based on experience. Randomized studies on treatment options are rare, due to the low prevalence of the disease as well as its heterogeneous clinical presentation. Many therapeutic options are supported by case reports. Treatment options are highly specific, and applicability in patients is often dependent on mutational status and clinical features. MC cannot be eradicated, though cytoreductive therapies can reduce the MC load.

Features in mastocytosis are caused by inappropriate MC activation and infiltration of MCs in various tissues. Treatment intervention targeting MC activation consists of three main aspects.

First, patient-specific triggers that may provoke MC activation should be avoided. Patient tolerability to nonsteroidal anti-inflammatory drugs (NSAIDs) or opioids should be tested prior to prescription, and if necessary, the use of these drugs must be avoided. When opioids are indicated, fentanyl is preferred over morphine [25]. Many anaesthetic agents can trigger MC degranulation and hence anaphylactic reactions. Therefore, patients with mastocytosis should be regarded as high-risk patients. Prior to procedures requiring the use of general anaesthesia or contrast media, patients should be pre-medicated with antihistamines and glucocorticosteroids. Anaesthesia should be induced preferably by propofol, etomidate, ketamine, cisatracurium or pancuronium. Maintenance can be achieved by a total intravenous technique or with a volatile anaesthetic such as sevoflurane [16•]. Several other triggers are known to elicit MC degranulation, including emotions, stress, alcohol, heat and physical stimuli such as friction or pressure.

Second, if possible, specific trigger treatment should be initiated. Mastocytosis patients generally have a higher risk of anaphylactic reaction. The life-threatening nature of anaphylaxis and the impact on QoL from fear of a reaction make the prevention of anaphylaxis an important aspect of treatment of mastocytosis patients. It has been recommended that all mastocytosis patients be provided with an adrenaline auto-injector. At least two devices should be provided to mastocytosis patients suffering from hymenoptera venom anaphylaxis, given the life-threatening nature of these reactions [26]. In addition, venom immunotherapy (VIT) has been recommended to reduce the likelihood of anaphylactic reactions. In order for VIT to be effective in mastocytosis patients, it should be performed as a lifelong treatment. Furthermore, VIT in mastocytosis is more often accompanied by side effects than in the general insect venom allergy population [27]. Based on the natural course of mastocytosis, we question whether VIT should even be considered in patients with increased IgE levels who have never experienced an allergic reaction due to hymenoptera stings, since fatal anaphylactic reactions have also been reported here [28].

Third, treatment to block the release of MC mediators is indicated. These types of treatment control MC mediator production as well as their mechanism of action. Treatment is highly individualized. The large heterogeneity in mutational patterns results in a heterogeneous composition of released mediators and hence a heterogeneous clinical presentation. The various symptoms require the appropriate therapeutic approach. The advantage of this type of treatment is that it is associated with relatively few side effects. Unfortunately, its effectiveness is not satisfactory in all patients.

**Fig. 1:**
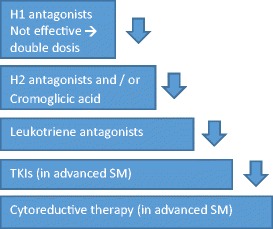
Add-on steps for mastocytosis treatment. Start treatment with H1 antagonists. Add H2 antagonists and/or cromoglicic acid in the case of GI symptoms or inadequately resolved pruritus. Leukotriene antagonists are added in the case of musculoskeletal pain or inadequately resolved pruritus. Tyrosine kinase inhibitors are thus far used only in patients with advanced SM.

Therapeutic options can be subdivided into antihistamines, leukotriene antagonists, cromoglicic acid systemic glucocorticosteroids, tyrosine kinase inhibitors (TKIs) and cytoreductive treatment. A combination of drugs is often necessary to achieve symptom control. An overview of the add-on steps in therapeutic options can be seen in Fig. 1.

Antihistamines are advised as a starting point in all mastocytosis patients. Even patients with no apparent symptoms may experience an improvement in their QoL after administration of antihistamines—for example, improved concentration or reduced foggy sensation in the head. Both H1 and H2 antihistamines block specific receptor-mediated symptoms as well as mutual activation of specific MC receptors. H1R are effective in diminishing symptoms like flushing, pruritus, wheals, swelling and abdominal cramping, whereas H2R are effective in reducing peptic GI symptoms. Second- and third-generation H1 antihistamines are preferred over first-generation agents because of their less sedating nature. Our personal preference is levocetirizine, given its minimally sedating character and good clinical response. Maximum dosage is 20 mg daily, administered in two doses. Ranitidine is a frequently prescribed H2 antihistamine.

Leukotriene antagonists block the action of leukotrienes. Montelukast, an antagonist of the cysteinyl leukotriene receptor 1 (Cys-LT1), and zileuton, a 5-lipoxygenase inhibitor (not available in Europe), are effective in treating prostaglandin-mediated symptoms such as flushing. They are also effective in reducing pruritus and musculoskeletal pain. Montelukast has been shown to reduce symptoms in children as well [29]. Leukotriene antagonists seem to improve the efficacy of H1 and H2 antagonists.

Cromoglicic acid is an MC stabilizer that acts through the G protein-coupled receptor (GPR)35. It is mainly of use in treating gastrointestinal symptoms, including diarrhoea, abdominal cramping, vomiting and meteorism [30]. Gastrointestinal resorption is negligible. A phase II trial evaluating the effects of dispersion (PA101) in the lungs is currently under way, with results expected by the end of 2016 [31]. Phase I results showed relevant serum concentrations with this type of administration (own data).

Systemic glucocorticosteroids are used in the treatment of acute anaphylactic reactions and in patients suffering from pulmonary symptoms such as wheezing.

TKIs have been used thus far only in patients with advanced SM. Most have shown little effectiveness, or their effect has been restricted to a small subgroup of mastocytosis patients. In the future, they may also be of use in patients with ISM, though more data on their effectiveness and safety in these patients are needed.

Imatinib therapy was shown to elicit a marked clinical response in patients without a KIT D816V mutation, but not in patients with a D816V mutation. Therefore, it is not appropriate for the majority of mastocytosis patients.

Studies on the suitability of dasatinib as a potential treatment option showed poor results. Masitinib did appear to improve symptoms in some SM patients [4].

A new treatment option is the TKI midostaurin (PKC412), an orally active small molecule that inhibits multiple kinases, including wild-type and mutant KIT D816V. Midostaurin was very recently shown to be highly effective in patients with advanced systemic mastocytosis (SM-AHNMD, ASM, MCL) [32••], with an overall response rate of 60 %, regardless of subtype. Forty-five percent of patients demonstrated a major response, meaning complete resolution of at least one type of mastocytosis-related organ damage, while 15 % showed a partial response. A phase II study on the effectiveness of midostaurin in patients with ISM is currently being executed in our centre. Data on symptom improvement are very promising.

Cytoreductive therapies, such as interferon alpha (IFN-α) and cladribine (2-CdA), are sometimes used as a therapeutic option in advanced systemic mastocytosis, in light of the aggressive course and poor prognosis of this disease. In ISM, cytoreductive therapy is not indicated, given the normal life expectancy of patients and the extensive side effects and mutagenic nature of cytoreductive drugs [33].

IFN-α and 2-CdA are non-targeted cytoreductive therapies, and have shown some effect in several studies, especially amongst patients with slowly progressing ASM. In patients with rapid disease progression, however, IFN-α response was usually absent. Response to 2-CdA was also limited in rate and duration [34].

In MCL, progression and symptoms may be so severe that allogenic hematopoietic cell transplantation (alloHCT) is required. A retrospective study has reported that alloHCT may be a potentially curative therapeutic option for advanced SM [35]. This should be done only after debulking with polychemotherapy or repeated cycles of 2-CdA [34].

Hydroxyurea is used as palliative cytoreduction if patients have no response to polychemotherapy or 2-CdA.

A recent study explored tamoxifen citrate as a cytoreductive treatment option in ISM patients. Tamoxifen was well tolerated by patients, but it did not reduce their MC load [36].

Studies are also investigating targets other than KIT, such as the PI3-kinase-AKT pathway [4]. This has shown positive results in vitro, though no effect has yet been found in vivo [37].

## Treatment options can also be listed per clinical complaint

Recovery from acute anaphylactic reactions is established using epinephrine, administered as either an intramuscular or an intravenous injection. Antihistamines, corticosteroids and omalizumab may have some additional effect.

Gastrointestinal complaints are treated with H2 antihistamines, cromoglicic acid, proton pump inhibitors, leukotriene antagonists or ketotifen.

Neurological symptoms may respond to antihistamine treatment. Cromoglicic acid and ketotifen have also been reported as treatment.

Cardiovascular and pulmonary symptoms are also initially treated with antihistamines. If this is not sufficient, corticosteroids can be effective. Pulmonary symptoms can be also be treated with leukotriene antagonists, and cardiovascular symptoms may show improvement on omalizumab.

Naso-ocular symptoms are treated with topical formulations of H1 antihistamines, corticosteroids and cromoglicic acid [38•].

Osteoporosis or other forms of bone involvement are a common feature in patients with mastocytosis. Osteoporosis is normally treated according to the guidelines for the general osteoporosis population, which focuses on increasing bone mineral density (BMD) in order to prevent fractures. Treatment typically consists of calcium and vitamin D supplements, often complemented with bisphosphonates, depending on osteoporosis severity and fracture risk. Considering the differences in pathophysiological mechanisms between mastocytosis and general osteoporosis, it is questionable whether the mechanism of action of bisphosphonates—increasing BMD—is effective in preventing fractures in mastocytosis patients. Thus far, the effectiveness of bisphosphonate treatment has been investigated only in small studies [39•, 40–44]. They have shown relatively good results, with a low prevalence of fractures and an increase in BMD during follow-up, but they are limited by low numbers of participants and/or short follow-up duration. We are currently conducting a larger study on this subject, with a higher number of participants and a longer follow-up period.

Since pathological fractures are considered a C-finding in mastocytosis patients, several studies have reported on the use of cytoreductive therapy, IFN-α, in these patients [41, 43]. However, osteoporosis is a frequent finding in ISM patients, and pathological fractures are often reported in such patients with otherwise low disease activity. Opinions vary on whether a more aggressive approach is permissible in these patients, given the high occurrence of side effects with this type of treatment.

## Conclusion and future perspectives

Mast cell clonal disorders are a heterogeneous group of conditions, of which systemic mastocytosis is most important. Mastocytosis varies in presentation and severity, making it a relevant condition for multiple fields of medicine and requiring a multidisciplinary approach. Patients often have a somatic D816V mutation that causes the accumulation and activation of MCs. Therapy should be individualized, based on the severity, symptoms, triggers and response to therapy. Patients with ISM have a good prognosis, and thus cytoreductive therapy is not indicated. Therapy is aimed at reducing symptoms and treating secondary conditions such as osteoporosis.

In treating advanced SM, several treatments are available, none of them curative. The results from the phase II trial of the multikinase inhibitor midostaurin are promising with respect to improving symptoms and quality of life in advanced SM patients. Clinical trials in ISM patients are currently under way. In addition to drugs targeting KIT, efforts are being made to develop drugs for alternative targets, which may provide other treatment options in the future.

Studies investigating differences in genetic composition within subtypes of mastocytosis may be useful for finding additional treatment options for mastocytosis, since they may provide us with greater insight into the emergence of specific symptoms in specific patients.
